# Remote learning and students’ mental health during the Covid-19 pandemic: A mixed-method enquiry

**DOI:** 10.1007/s11125-020-09530-w

**Published:** 2021-01-05

**Authors:** Suzanne Lischer, Netkey Safi, Cheryl Dickson

**Affiliations:** Lucerne University of Applied Sciences and Arts–Social Work, Werftestrasse 1, 6002 Lucerne, Switzerland

**Keywords:** Remote learning, Covid-19, Stress, Mental health, Switzerland

## Abstract

The disruption caused by Covid-19 in the educational sector may last longer than originally predicted. To better understand the current situation, this article analyses the mental health status of university students during the pandemic and investigates the learning conditions needed to support students. The sample included 557 undergraduate students who took part in an online survey. Overall, the students reported coping well during lockdown but indicated that lecturers were challenged by distance teaching, which created some stress for the students.

In light of rising concern about the current Covid-19 pandemic, a growing number of universities across the world, beginning from March 2020, either postponed or cancelled all such campus events as workshops, conferences, sports (both intra- and inter-university), and other activities. Universities moved rapidly to transfer various courses and programmes from face-to-face to online teaching (Sahu 2020). Due to the continuing sharp rise in the number of infections, the Swiss Federal Council declared the “extraordinary situation” as early as March 16, 2020, passing an ordinance that placed massive restrictions on public life. Primary schools as well as universities had to close immediately. Since June 8, Swiss universities were able to resume their teaching activities, under the condition that they apply strict security measures; however, teaching will remain restricted for an unlimited period. Colleges and universities are thus facing decisions about how to continue teaching while keeping their faculty, staff, and students safe from a deadly virus emergency that is moving fast and not well understood (Hodges et al. 2020).

Within this context, the question arises as to how to tackle the latent threat of Covid-19. As pharmaceutical interventions such as vaccines are on the horizon but not yet available, universities, even after the end of lockdown, must apply nonpharmaceutical interventions, including social and physical distancing to slow the spread of the disease and prevent the emergence of new diseases. Having been thrust into online learning, literally overnight, academics have been debating what will become the “new normal” for our institutions and teaching practices, and what is the best way forward (Tesar 2020).

On the one hand, the potential of digital technologies to enhance student learning had already been well established before the outbreak. In the last few years, much enthusiasm has surrounded the development of digital technologies along increasingly personalised, remote, adaptive, and data-driven lines. The concept of blended learning to combine the benefits of face-to-face and online teaching is gradually being integrated into institutions (Dziuban et al. 2018), and digital technologies are clearly integral to the future of university education around the world (Henderson et al. 2017). Faculty training to support this transition—as well as student engagement and connectedness—has been identified as crucial to its success (Barr and Miller 2013). Whilst the use of already-familiar applications, such as WhatsApp, proved useful for remote-learning during the early pandemic (mid-March), research has highlighted the need for more effective practices for the post-emergency stage (Wargadinata et al. 2020). However, due to the sudden emergence of Covid-19, most faculty members faced unforeseen challenges, including lack of online-teaching experience, lack of time for preparing distance-learning courses, and figuring out how to make use of support from educational technology teams (Bao 2020). Thus, students and teachers faced problems when studying and teaching at home. Literature highlights certain deficiencies, such as the weakness of online-teaching infrastructures, the inexperience of teachers regarding new technologies, the information gap, the complex environment at home, and so forth (Ali 2020). Furthermore, students have a wide range of distractions when studying at home. For example, not all are able to find suitable spaces for home learning, or studying may be constrained by insufficient hardware and unstable networks (Zhang et al. 2020).

Findings suggest that students, as well as the general population, may be experiencing psychological effects from the outbreak of Covid-19, such as anxiety, fear, and worry, among others (Cao et al. 2020; Li et al. 2020; Wang et al. 2020). A longitudinal study reveals that, compared to prior academic terms, individuals in the Winter 2020 term were more sedentary, anxious, and depressed. In addition, a wide variety of behaviours, including increased phone usage, decreased physical activity, and fewer locations visited, are associated with fluctuations in Covid-19 news reporting (Huckins et al. 2020). Findings from China in February 2020 indicate that college students’ anxiety regarding the pandemic was associated with their place of residence, source of parental income, whether living with parents, and whether a relative or an acquaintance was infected with Covid-19 (Cao et al. 2020). Some students might be at higher risk of social isolation and the development of mental health problems during the Covid-19 crisis. This is particularly true when they live by themselves, have less direct contact to close family members and friends, receive less social support, and are less well-integrated into a social network of students. Female students appeared to be at higher risk of facing negative mental health consequences (Elmer et al. 2020).

## Aim

The disruption caused by Covid-19 in the educational sector may last longer than expected if a reliable solution for the virus is not found quickly and its spread continues. In this study, we aim to highlight the potential impact of the Covid-19 outbreak on the education and mental health of university students. We investigated and analyzed the mental health status (in particular, anxiety) of university students during the pandemic for the following purposes: (1) to evaluate these students’ emotional situation during the pandemic; (2) to find out what learning conditions that university management and lecturers should establish in order to provide the best possible support for students during this pandemic or a future epidemic/pandemic.

In this article we examine the following questions:

- How do students at the Lucerne University of Applied Sciences and Arts perceive the university’s support during the coronavirus pandemic?
- Which sociodemographic characteristics are associated with increased anxiety?
- To what extent were the students distressed—both psychologically and regarding the study workload—during the outbreak?
- Which Covid-19–related stressors are correlated with anxiety during the pandemic?
- What are, in the students’ views, the challenges and benefits of integrating distance learning in tertiary institutions, in response to the Covid-19 crisis?

## Method

We launched the survey for this cross-sectional study on April 23, 2020 and received responses through the end of May 2020. We designed and conducted the survey using the Enterprise Feedback Suite (EFS) Survey by Questback. The Central Switzerland Ethics Committee approved this study.

### Study population and sample

The study population is all undergraduate students at the Lucerne University of Applied Sciences and Arts is N = 5,200, across six faculties. The relevant departments informed the students about the survey in various ways (email, department website, and/or wider university website).

A total of 557 students took part in the survey. All participants voluntarily gave their informed consent to participate after being informed about the purpose of the study. The questionnaires were anonymous to ensure confidentiality and the reliability of data.

### Variable specification

#### Sociodemographic variables

We asked participants to report on their *gender* (male or female), *age group* (18–24, 25–34, 35–44, or 45–55 years), *nationality* (Swiss or non-Swiss), *migration background* (yes or no), *discipline* studied*, living situation* including *relationship status* (4 categories; see Table 1), *number of children*, and *household type* (6 categories; see Table 1). For the purposes of the analysis, the variable *living situation* was ultimately dichotomized into categories for cohabiting and for living alone in the community.

**Table 1:** Demographic characteristics of the sample and associations with PHQ-4 scores

		Total sample		PHQ4		Group differences	
		n	%	M	SD	*P* value	Effect size^a^
Gender	Female	286	63.8	1.9	0.7	.002	d = 0.31
	Male	162	36.2	1.7	0.6		
Age group	18–24	252	45.7	1.8	0.6	*n.s.*	
	25–34	236	42.8	1.9	0.7		
	35–44	46	8.3	1.7	0.7		
	45–54	18	3.3	1.5	0.4		
Nationality	Swiss	489	88.9	1.8	0.6	*n.s.*	
	Non-Swiss	61	11.1	2.0	0.8		
Migration background	No	484	89.7	1.8	0.6	.015	d = 0.25
	Yes	54	10.1	2.1	0.8		
Discipline	Social Work	225	40.4			*n.s.*	
	Engineering & Architecture	190	34.1				
	Information Technology	3	.5				
	Art & Design	104	18.7				
	Music	11	2.0				
	Business	24	4.3				
Relationship status	Spouse/partner, living in the household	148	29.9	1.9	0.8	*n.s.*	
	Spouse/partner living outside the household	125	25.3	1.8	0.6		
	No spouse/partner	169	34.1	1.8	0.6		
	Another form of relationship	53	10.7	1.8	0.6		
Number of children	No children	486	92.2			*n.s.*	
	1 child	24	4.6				
	2 children	11	2.1				
	3 children	6	1.1				
Household type	Single-person household	53	10.0	1.9	0.7	.032	η^2^ = .027
	Couple with no children	113	21.3	1.9	0.8		
	Couple with child(ren)	56	10.5	1.5	0.5		
	Single-parent household with child(ren)	70	13.2	1.8	0.7		
	Non-family household with several people (e.g., shared house)	161	30.3	1.8	0.6		
	Other	78	14.7	1.7	0.7		

^a^Effect sizes defined as follows: “small, d = .2, η^2^  = .02”, “medium, d = .5, η^2^  =  .13”, “large, d = .8, η^2^  = .26”

*Anxiety*. In addition to sociodemographic questions that addressed the students’ situation during the Covid-19 pandemic, the survey included a validated anxiety scale. The 4-item Patient Health Questionnaire-4 (PHQ-4) is a rapid self-reported measure. Respondents rate their symptoms using a 4-item Likert rating scale ranging from 1 (not at all) to 4 (almost every day), and the total score ranges from 0 to 16 (Löwe et al. 2010). We used Cronbach’s α (Cronbach 1951) to measure the scale’s reliability—the internal consistency. The PHQ-4 is a well-validated screening instrument, demonstrating a high internal consistency (Cronbach’s ɑ = 0.81). The scale categorises the severity of clinically relevant depression and/or anxiety according to the PHQ-4 score, as follows: normal (1–4), mild (4–8), moderate (9–12), severe (13–16).

*Covid-19–related stressors.* We assessed Covid-19–related stressors using a set of questions. which were constructed by a group of researchers at the University Hospital Frankfurt. The first question, operationalized into 3 items, examined what effects students feared might occur as a result of the Covid crisis: *Worry about economic impact*; *worry about loss of social contact;* and *worry about academic delays*. A 3-point scale was used to rate each item, as follows: *So far, it is not an issue* (1); *that is what I fear* (2); *has already happened* (3) (Frankenberg et al. 2020b).

The second question examined concerns about health and the social environment. The survey asked students about the statements*: I worry over personal health issues* (becoming depressed); and, *I worry about health issues for people close to me* (e.g., parents, grandparents). Respondents reported their answers using a 4-item Likert rating scale ranging from *strongly disagree* (1) to *strongly agree* (4) (Frankenberg et al. 2020a).

*Experiences with remote studying during the Covid-19 pandemic.* We asked open-ended questions about participants’ experiences with home study during the outbreak, inviting the students to report on the challenges and advantages of home study. In addition, we asked them how they perceive the support given by the university and what kind of response they would like to receive.

#### Data analysis

We analyzed data using SPSS Version 22.0. We conducted an analysis of the descriptive statistics to illustrate demographic and other selected characteristics of the respondents (e.g., students’ self-reported anxiety levels) and used a one-way analysis of variance (ANOVA) to compare the means (PHQ-4 scores) between different groups (e.g., gender). We ran a regression analysis to explore the significant associations between Covid-19–related stressors (worry about: economic impact, delays in academic activities, loss of social contact, own health, and health of others) and different levels of self-reported anxiety (normal, mild, moderate, severe). We employed MAXQDA 2020 to analyze the students’ qualitative responses concerning home-study experiences during the outbreak.

## Results

In Table 1, we present the demographic and selected characteristics of the study population. Among the sample of 557 undergraduate students who took part in the survey, from a total of six faculties, 448 provided information on their gender. The majority of respondents were female, and the mean age was 27 (median, 25). The response rate from the different faculties varied considerably: Social Work (40.4%), Engineering and Architecture (34.1%), Information Technology (0.5%), Art and Design (18.7%), Music (2.0%), and Business (4.3%).

### Anxiety levels

#### Factors influencing anxiety levels: Univariate analysis (ANOVA)

The findings indicate that women had significantly higher mean anxiety scores (PHQ-4) when compared to men (F[1, 446] = 9.661, *p* = .001), respectively. However, the effect size was small (*p* = .002, Cohen’s *d* = .319). We observed no significant difference in mean anxiety scores with regard to age (*p* = .057); also, there was no group difference in mean anxiety scores regarding nationality (Swiss vs. non-Swiss). Regarding migration background, students who had not migrated had significantly higher mean anxiety scores when compared to those who had migrated (F[1, 443] = 8.501, *p* = .015). This effect was also very small (*p* = .015, *d* = 0.25). Differences in relationship status and number of children were not related to the PHQ-4 mean scores. Couples with children had significantly lower average anxiety values than single-person households, couples without children, etc. (F[5, 447] = 2.468, *p* = .032, η2 = .027).

In conclusion, it appears that an increased PHQ-4 score is not related to certain sociodemographic characteristics. The differences found (gender, age, migration background, type of household) have no significant effect.

Table 2 shows how the mental health of college students was affected to varying degrees during the outbreak. Of the responding students, 85.8% reported experiencing anxiety, for which the symptoms, in the majority of cases, can be classified as “mild”.

**Table 2:** Students’ self-reported anxiety levels (n = 458)

Anxiety level	n	Ratio (%)
Normal	65	14.2
Mild	290	63.3
Moderate	75	16.4
Severe	28	6.1
Total	458	100

#### Correlation between Covid-19–related stressors and PHQ-4 anxiety scores

We show, in Table 3, the results of the regression analysis between Covid-19–related stressors and PHQ-4 anxiety levels. Concerns about the economic impact of the pandemic were positively related to the college students’ levels of anxiety (*r* = 0.117, *P* < .05). Moreover, concerns about academic delays (*r* = 0.135, *P* < .01) or over personal health issues (becoming depressed) (*r* = 0.194, *P* < .001) and worry about health issues for close others (e.g., parents, grandparents) also positively correlate (r = 0.171, P < .001) with the level of anxiety. Worry about loss of social contact was not correlated with anxiety levels (r = .073, *P* = .132).

**Table 3:** Regression analysis of COVID-19-related stressors and self-reported anxiety levels (PHQ-4)

Related stressor	Anxiety level	
	*R*	*P*
Worry about economic impact	.117	.033
Worry about academic delays	.135	.006
Worry about loss of social contact	.073	.132
Worry about personal health issues (becoming depressed)	.194	.000
Worry about health issues for close others (e.g., parents, grandparents)	.171	.000

### Students’ experiences of distance learning

As part of the open-ended questions about students’ experiences of distance learning, we asked them to report on what challenges and what opportunities distance learning brings in response to the Covid-19 pandemic. N = 370 of 557 students (66%) answered the question “What works well [regarding your current challenges in distance learning]?”; and 406 students (72.9%) answered the question “What are the difficulties?”.

As the following results show, the students handled the new situation pragmatically. While some students tackled the work with a high degree of discipline and explicitly emphasized the advantages of the increased personal responsibility and independent working, (n = 82), others expressed difficulties in concentrating and maintaining the necessary motivation for distance learning (n = 76). For some students (n = 24), time management was a particular challenge. Structural conditions, such as having only one room serving as both bedroom and study, complicated the situation. Eight students stated that they had difficulties with the limited available space.

Even though the students had already dealt with IT before Covid-19, the abrupt change to exclusively digitalized communication channels was a challenge. Students did not cope equally well with the technical requirements. Some saw the setup of the different IT tools as complex. Whilst some students emphasized that the technology worked perfectly (n = 17), other students complained about technical problems (n = 10), such as the slow internet connection. Overall, however, they assessed the tools (e.g., for the learning platform or video communication) as practical (n = 24). In particular, they very much appreciated the use of zoom for providing the classroom and for bilateral discussions (n = 57). However, they sometimes perceived group discussions via zoom as sluggish (n = 10).

If teachers give distance-learning work assignments, it is important from the students’ point of view that these are given at an early stage, that the assignment is clear, and that they know what further steps may be needed. Furthermore, the opportunity to clarify ambiguities must be provided, and the required workload must be proportionate. A total of n = 23 students emphasized that the work assignments were didactically meaningful and that the lecturers adequately communicated them. The performance of the lecturers received mixed reviews, though this is also the case for face-to-face classes. For example, the fact that teachers uploaded PowerPoint presentations to the learning platform without setting them to audio was seen as unhelpful (n = 11); n = 17 students negatively appraised the unstructured uploading of teaching materials to the learning platform Ilias. Group work in distance learning was described as challenging and sometimes unnecessary. Students expressed their wishes that this learning method be used carefully and purposefully in a digital environment (n = 39). Students' comments included:

Every lecturer has a different idea of how distance learning should work. A grid would help. Ideally, for example, this grid structure would be useful: In the run-up to the course, students prepare themselves by reading, then the topic is explained in an online sequence by a lecturer. Afterwards, there should be an opportunity to clarify questions and discuss the issue.

Work assignments are delivered in advance, there are forums available for questions.

Clear assignments, I can easily, sometimes even more simply, acquire most of the module contents in Distance Learning.

However, n = 11 students were critical about their experience. In particular, the extra workload and the partly unclear communication caused the students problems. Comments were related to: increased planning effort to coordinate video conferences (different tools and different information channels are used), extra deadlines due to the changeover to distance learning, extensive self-study without additional explanation. The fact that different learning platforms are used was perceived as frustrating. In this context, students also discussed the digital competence of the lecturers, which they perceived as varying widely. One said:

Zoom meetings are tiring but good for discussions, some of the lecturers lack digital literacy.

Sixteen students reported uncertainties about exam preparation. Not surprisingly, students explicitly welcomed the university’s concession that they would not, in any circumstances, be marked as having “failed” the exam and that examination forms were adapted to take into account the actual teaching they had received.

Respondents’ discussion reflected controversy over communication on the part of the university management. Overall, negative feedback with regard to communication predominated (n = 16). In particular, students evaluated critically the *“flood of emails and information”* to which they were exposed; they saw lecturers’ email communication as uncoordinated in some cases. Eleven students stated that they missed the direct exchange with the lecturers and, in particular, concrete feedback on work assignments that they had done.

A total of n = 51 students stated that they lacked personal exchanges with fellow students. However, they also noted that, on occasions where such exchanges had been possible, these had been highly appreciated (n = 10).

The overall conclusion is that the majority of students would like to see a return to face-to-face classes. N = 50 students were critical of Covid-19 conditional distance learning and would like to see a return to the teaching methods used before the outbreak. At the same time, 42.5% of the students agreed with the statement that a switch to higher levels of distance learning should also be targeted for the period after the Coronavirus pandemic.

## Limitations and future directions

The current study has several limitations that could be addressed within future research. Firstly, of the approximately 5200 students at the university, only 557 students across all departments took part in the survey. This is a moderately low response; the results are therefore not representative. Second, students in some fields, such as music or computer science, are barely represented in the sample. In view of the fact that the area of study plays a very important role in the discussion about the advantages and disadvantages of distance learning, this is a major constraint of this study. The different response rates can be explained by the fact that invitations to participate in the study were distributed in different ways. The Departments of Social Work, Engineering and Architecture, and Art and Design sent the invitation by email with the survey link, while the Departments of Information Technology (IT), Music, and Business only posted it on the department’s website or on the internal website, without explicitly referring to the survey. Moreover, we did not find strongly significant effects on mental health. However, the exploratory evaluation using open-ended questions raises useful issues for further research work, such as whether student satisfaction will increase once streamlined digital processes and personalized support measures are fully integrated.

## Conclusions

The Covid-19 outbreak has disrupted the lives of many people across the world. The rapid increase in cases of infection, worldwide, has created uncertainty and anxiety about what is going to happen. It has also caused a tremendous level of stress among students. Previous studies have suggested that public health emergencies such as the Covid-19 pandemic can have many psychological effects on college students, which can be expressed as anxiety, fear, and worry, among others (Cao et al. 2020; Huckins et al. 2020; Li et al. 2020; Wang et al. 2020). This stress may lead to unfavourable effects on the learning and psychological health of students (Sahu 2020).

Whilst not designed to be representative, the survey aimed to gain insight about potential impact of the Covid-19 outbreak on the education and mental health of undergraduate students. The survey gathered n = 487 responses from undergraduate students who are studying in one of the 6 departments of the Lucerne University of Applied Sciences and Arts. Regarding anxiety and stress, the study reveals that 85.8% of the students reported symptoms of anxiety, although in the majority of cases these symptoms were mild (63.3%). The study did not confirm previous findings that students who live alone are at higher risk of developing mental health problems (Elmer et al. 2020). However, female students appeared to be at higher risk of facing negative mental health consequences, even though the effect size is small. Due to the cross-sectional nature of this study, we cannot know whether these symptoms existed before the pandemic. Yet, there are several plausible explanations for why students may feel stressed or anxious during the Covid-19 outbreak, including additional study-related uncertainties or worries about career prospects.

Nevertheless, the results of the survey suggest that the students coped well with stresses that occurred during the lockdown. Moreover, the majority of the students felt well supported and expressed their appreciation of the lecturers. However, this is no reason for either the university management or the lecturers to rest on their laurels. The results of the open-ended questions indicate that distance teaching was a challenge for lecturers, which in turn created stress for the students. Thus, perhaps more than anxiety and stress, the experience of a rapid online transition to remote teaching has revealed much about the deficiencies of the higher education sector and, perhaps, much about what needs to change in universities (Watermeyer et al. 2020). Lecturers as well as students have to be prepared for future times that require flexibility and probably a higher workload, and greater effort in order to study. Digital literacy is no longer a “nice to have” but dispensable competence for both lecturers and students. There are many reasons to believe that Covid-19 has created “a new normal” for the universities—one that will continue after the lockdown ends. The rapid evolution of Information Communication and Technology (ICT) and the increasing complexity that comes with its vast potential explains why integration of technology in education continues to receive special attention, particularly in the wake of the Covid-19 pandemic (Ali 2020). It is up to university management to provide both lecturers and students with the necessary tools to acquire these competences.
